# Workflow of CAD / CAM Scoliosis Brace Adjustment in Preparation Using 3D Printing

**DOI:** 10.2174/1874431101711010044

**Published:** 2017-10-24

**Authors:** Hans-Rudolf Weiss, Nicos Tournavitis, Xiaofeng Nan, Maksym Borysov, Lothar Paul

**Affiliations:** 1 Orthopedic Rehabilitation Service, Alzeyer Str. 23, D-55457 Gensingen, Germany; 2 Scoliosis Best Practice Rehab Services, Thessaloniki, Greece; 3 Nan Xiaofeng's Spinal Orthopedic Workshop, Beilin district of Xi'an Shaanxi, China; 4 Orttech-plus Rehabilitation Service, Ukraine, Kharkiv; 5 GFaI, Volmerstraße 3, 12489 Berlin

**Keywords:** Scoliosis, Brace treatment, CAD / CAM, 3D printing, Additive manufacturing, Correction, Comfort, Outcome

## Abstract

**Background:**

High correction bracing is the most effective conservative treatment for patients with scoliosis during growth. Still today braces for the treatment of scoliosis are made by casting patients while computer aided design (CAD) and computer aided manufacturing (CAM) is available with all possibilities to standardize pattern specific brace treatment and improve wearing comfort.

**Objective:**

CAD / CAM brace production mainly relies on carving a polyurethane foam model which is the basis for vacuuming a polyethylene (PE) or polypropylene (PP) brace. Purpose of this short communication is to describe the workflow currently used and to outline future requirements with respect to 3D printing technology.

**Method:**

Description of the steps of virtual brace adjustment as available today are content of this paper as well as an outline of the great potential there is for the future 3D printing technology.

**Results:**

For 3D printing of scoliosis braces it is necessary to establish easy to use software plug-ins in order to allow adding 3D printing technology to the current workflow of virtual CAD / CAM brace adjustment. Textures and structures can be added to the brace models at certain well defined locations offering the potential of more wearing comfort without losing in-brace correction.

**Conclusions:**

Advances have to be made in the field of CAD / CAM software tools with respect to design and generation of individually structured brace models based on currently well established and standardized scoliosis brace libraries.

## 1 BACKGROUND

Scoliosis is a three-dimensional deformity of the spine and trunk, which may lead to significant health problems when left untreated [1]. During growth, such a deformity may progress within a short period of time [1, 2]. Therefore, according to the current guidelines, conservative treatment with high corrective exercises and high corrective braces are indicated to prevent such a spinal deformity from being progressive [3]. High correction bracing is the most effective conservative treatment for patients with scoliosis during growth. Many different braces are in the market nowadays, but not all with the best possible outcome [4], which would outweigh the impact of brace treatment in children and adolescent patients during growth. Boston brace treatment in a randomized controlled study has been proven effective with a success rate of 72% [5]. While the Boston brace is more or less symmetric with pads inserted leading to compression effects which might be experienced as being uncomfortable by the patients, more asymmetric braces using the Chêneau standard of pattern specific correction in a prospective controlled study have been shown to be able to improve the outcome with a success rate of 80% Fig. (**1**) [6]. In a more recent retrospective paper, success rates exceeding 90% have been achieved [7] while the last prospective cohorts using the recent developments of the Chêneau standard (Gensingen Brace^®^) had success rates of about 90% Figs. (**2** and **3**) [8, 9].

Still today, braces for the treatment of scoliosis are made by casting patients while computer aided design (CAD) and computer aided manufacturing (CAM) are available with all possibilities to standardize pattern specific brace treatment and improve wearing comfort [4]. The Boston Brace^®^ [5] today is the most widely used symmetric brace and the Gensingen Brace^®^ (GBW) [4] is the most widely used asymmetric brace for the treatment of scoliosis. The adjustment of the GBW is based on a classification of different curve patterns and on a library of specific brace models to address the different geometric deviations [4].

## 2 OBJECTIVE

CAD / CAM brace production mainly relies on carving (milling) of a polyurethane foam block which is the basis for vacuuming a polyethylene (PE) or polypropylene (PP) brace model. Purpose of this short communication is to describe the workflow currently used and to outline future requirements with respect to 3D printing technology.

## 3 METHOD

A workflow description of virtual brace adjustment as available today is the content of this paper as well as an outline of the great potential of 3D printing technology.

The basis for best possible brace adjustment is the availability of the patient’s record including sex, age, maturity, angle of curvature (Cobb angle), and the diagnosis. Additionally, an evaluation of the geometric entity of the deformation is required in order to find the appropriate curve pattern, facilitated by means of the patient’s pictures from all four sides and of the x-ray.

In principle, the brace can be adjusted virtually by using the hand measurements of the patients’ trunk, but a 3D-scan of the patient’s body is preferred. The virtual adjustment process described within this paper is based on the Final Surface^®^ software and on the customized ScoliCAD^®^ plug-ins developed for the adjustment of the GBW.

**Fig. (4):**
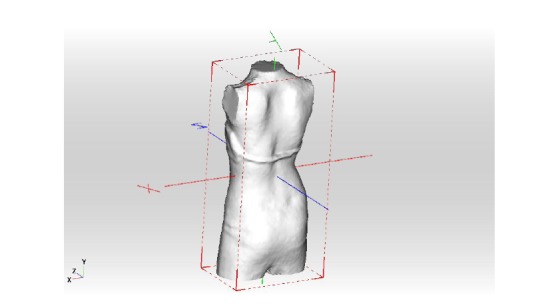
The patient’s scan has to be adjusted in a standard coordinate system.

1. The patient’s scan has to be adjusted in a standard coordinate system. This is necessary to derive parametric measurements from the 3D-scan for a raw adjustment of the brace (Fig. **4**)
2. The patient’s scan has to be scaled, which is a semi-automated process using the ScoliCAD^®^ Scaler. However this process has to be controlled and manually fine-adjusted in some cases Fig. (**5**).
3. The appropriate brace model must be chosen from the library and then inserted into the scene Fig. (**6**)
4. The brace model is then adjusted using the ScoliCAD^®^ Adjuster and the fine adjustment is made individually by hand Fig. (**7**).
5. With the help of the ScoliCAD^®^ Brace Designer the correction in frontal plane (shift) and sagittal plane is individually increased for each patient Figs. (**8** and **9**)

Currently the resulting brace model represents a negative mould of the brace to be manufactured. It can be exported as an STL-file to be carved at any milling service. The brace form is then milled from a PU foam block. This form in turn is used for vacuuming (thermoforming) the final brace, which can be adjusted to the patient’s body after being cut from the foam model and some adequate finishing steps. (The used PU foam body remains as waste and/or is subject to recycling.)

## 4 RESULTS

The results achieved with this technology seem to be better than the results published for cast based braces made completely by hand. For handmade Boston and Chêneau style braces, success rates were between 70 and 80% [5, 6], while the success rates with comparable samples fabricated in the described above standardized technology were about 90% [4, 7-9]. In order to improve the success rate further, brace wearing time besides in-brace correction seems to be of vast importance [10]. Therefore, it seems necessary to improve wearing comfort in order to achieve a better compliance of the patients.

3D printing [11] would offer the opportunity of making braces lighter and more comfortable by using alternative materials, by adapting wall thicknesses and individually structured designs including local textures, pierced or reinforced regions of the brace. The integration of such well-defined local textures and structures offers the potential of increased wearing comfort, particularly determined by lighter brace weight, more adjustable or flexible zones and better aeration without losing in-brace correction. (As a technological and environmental side effect, wasting / recycling large amounts of PU foam material would become unnecessary with 3D printing.)

However, the current software as of yet, does provide neither the spectrum of functions and tools nor the usability needed to create this kind of improved brace models. In order to add 3D printing technologies to the current toolbox of scoliosis brace manufacturing Fig. (**10**), it is necessary to develop and establish easy-to-use software tools in the already existent workflow of virtual CAD / CAM brace adjustment.

## CONCLUSION

It was concluded that advances have to be made in the field of CAD / CAM software with respect to

(1) the derivation of consistent directly printable (positive) brace models from both individual patient 3D-scans and established standardized scoliosis brace form libraries and

(2) easy-to-use tools for quick, individual redesign of certain brace regions targeting weight reduction, local stiffening, adaptivity and aeration.

With the optimal therapeutic form already defined, the technologically unlimited design options of 3D printing technologies offer huge potential for significant improvements in wearing comfort and patient compliance and finally the outcome of brace treatment for patients with spinal deformities.

Future options of 3D printing technology will go far beyond what is possible today. 3D printing is not just another way of fabricating standardized braces, but the key for the development of new brace generations connecting high correction success with improved wearing comfort features and higher patient compliance.

## Figures and Tables

**Fig. (1):**
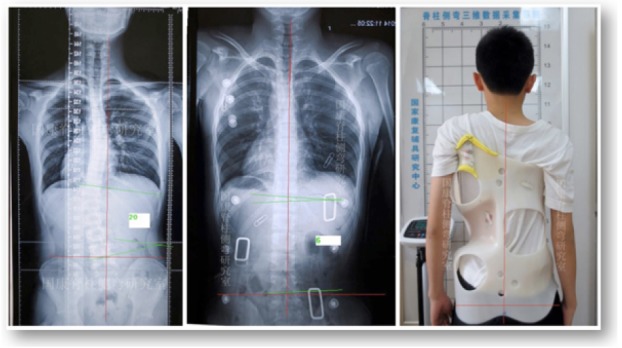
Cast based Chêneau brace with nearly full correction of a double major curve pattern. This ‚old style‘ Chêneau brace has been produced in China (with kind permission by Xiaofeng Nan, Xi‘an)

**Fig. (2):**
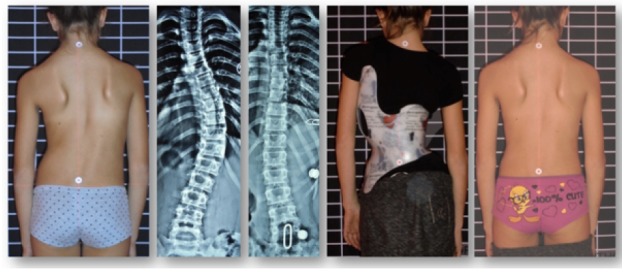
Gensingen Brace^®^ treatment for a single curve pattern showing an overcorrection in the x-ray and a slight cosmetic improvement after 6 weeks of brace wearing time. This Gensingen brace has been made in Greece (With kind permission by Nico Tournavitis, SBPRS, Thessaloniki, Athens, Nicosia).

**Fig. (3):**
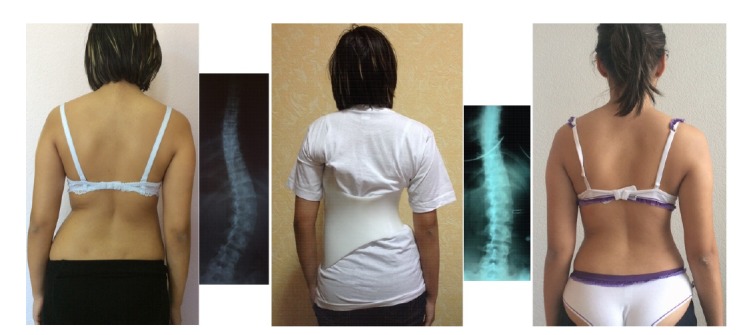
Gensingen Brace^®^ treatment for a single curve pattern of 50 degrees showing a good correction in the x-ray and a significant cosmetic improvement after 6 months of brace wearing time. This Gensingen brace has been made in Ukraine (With kind permission Maksym Borysov, Orttech-Plus Rehabilitation Service, Kharkiv).

**Fig. (5):**
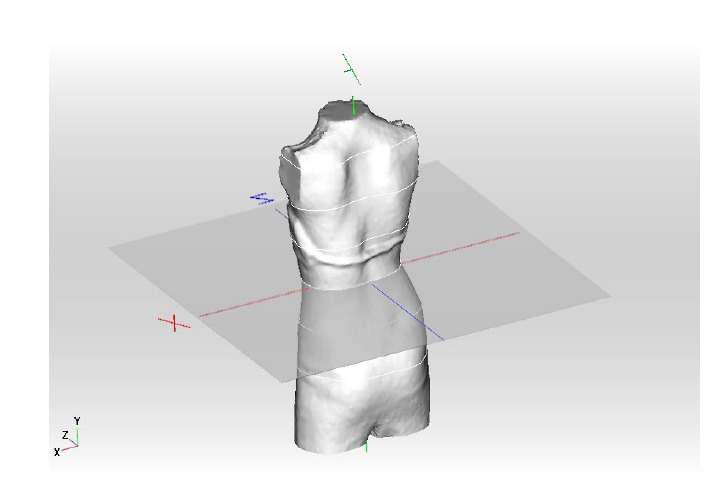
The patient’s scan has to be scaled, which is a semi-automated process using the ScoliCAD^®^ Scaler.

**Fig. (6):**
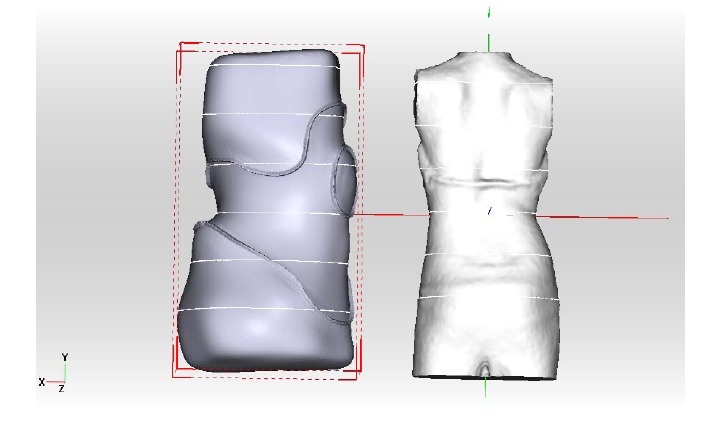
The appropriate brace model must be chosen from the library and then inserted into the scene.

**Fig. (7):**
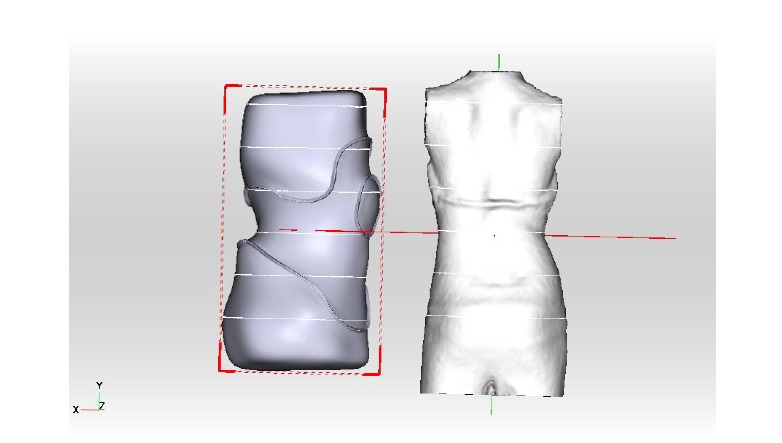
The brace model is then adjusted using the ScoliCAD^®^ Adjuster and the fine adjustment is made individually by hand.

**Fig. (8):**
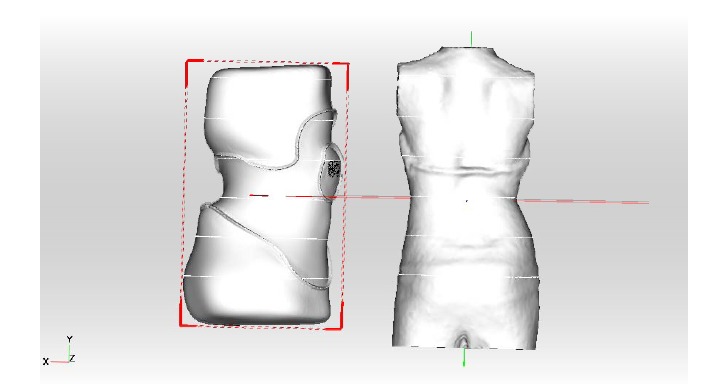
With the help of the ScoliCAD^®^ Brace Designer the correction in frontal plane (shift) and sagittal plane is individually increased for each patient.

**Fig. (9):**
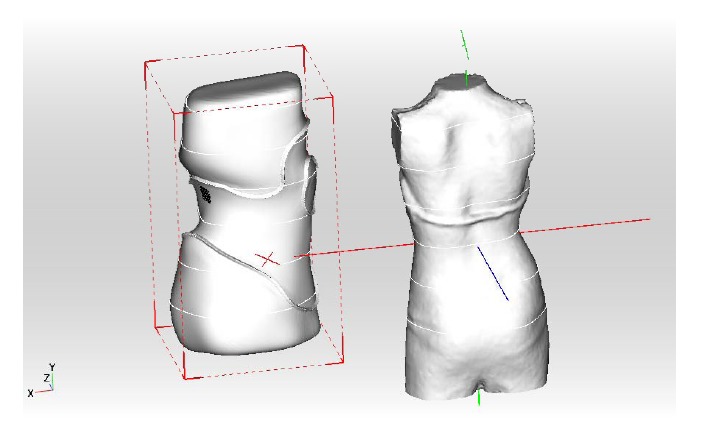
Before releasing the STL-file the final result of virtual brace adjustment needs to be reviewed from all sides.

**Fig. (10):**
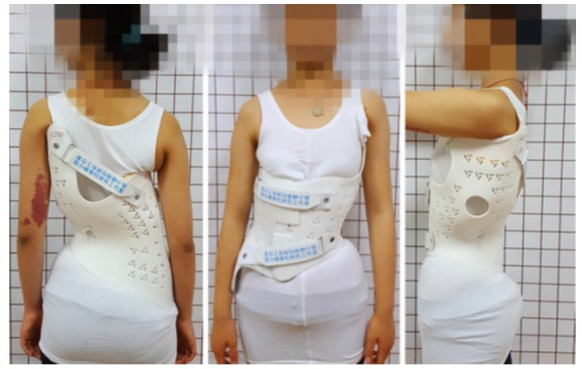
Chinese patient with a GBW as printed in China and adjusted by Xiaofeng Nan, Xi’an. This brace has been printed using SLS technology (selective laser sintering), which is slowly and costly but offers a wider range of materials to be printed. First braces currently are made on the basis of FFF (fused filament fabrication) which currently is cheaper and faster.
